# Left bundle branch block in Duchenne muscular dystrophy: Prevalence, genetic relationship and prognosis

**DOI:** 10.1371/journal.pone.0190518

**Published:** 2018-01-05

**Authors:** Abdallah Fayssoil, Rabah Ben Yaou, Adam Ogna, Cendrine Chaffaut, France Leturcq, Olivier Nardi, Karim Wahbi, Denis Duboc, Frederic Lofaso, Helene Prigent, Bernard Clair, Pascal Crenn, Guillaume Nicolas, Pascal Laforet, Anthony Behin, Sylvie Chevret, David Orlikowski, Djillali Annane

**Affiliations:** 1 Service de Réanimation médicale et unité de ventilation à domicile, CHU Raymond Poincaré, APHP, Université de Versailles Saint Quentin en Yvelines, Garches, France; 2 Centre d’Investigation clinique et Innovation technologique CIC 14.29, INSERM, Garches, France; 3 Institut de Myologie, CHU Pitié Salpetrière, Paris, France; 4 Sorbonne Universités, UPMC Univ Paris 06, INSERM UMRS974, Centre de Recherche en Myologie, Institut de Myologie, G.H. Pitié Salpêtrière, Paris, France; 5 Laboratoire de biochimie et génétique moléculaire, hôpital Cochin, AP-HP, université Paris Descartes-Sorbonne Paris Cité, Paris, France; 6 SBIM, CHU Saint Louis, APHP, Université Paris Diderot, Paris, France; 7 Service de Cardiologie, CHU Cochin, AP-HP, université Paris Descartes-Sorbonne Paris Cité, Paris, France; 8 Service de Physiologie—Explorations fonctionnelles, CHU Raymond Poincaré, APHP, Université de Versailles saint Quentin en Yvelines, Garches, France; 9 Service de médecine aigue, CHU Raymond Poincaré, APHP, Université de Versailles Saint Quentin en Yvelines, Garches, France; 10 Service de Neurologie, CHU Raymond Poincaré, APHP, Université de Versailles Saint Quentin en Yvelines, Garches, France; Rutgers University Newark, UNITED STATES

## Abstract

**Background:**

Duchenne muscular dystrophy (DMD) is an inherited myogenic disorder due to mutations in the dystrophin gene on chromosome Xp21.1. We designed this study to determine the prevalence of left bundle branch block (LBBB), whether there is a relationship between LBBB and genetic pattern, and to assess predictive factors for acute cardiac events and mortality in adult DMD patients.

**Methods:**

We reviewed the charts of DMD followed at the Home Mechanical Ventilation Unit of the Raymond Poincare University Hospital.

**Results:**

A total of 121 patients, aged from 18 to 41 years have been included in our study. Median vital capacity (VC) was 12% [7; 19.5] of predicted. Almost all patients were on home mechanical ventilation (95%). LBBB was present in 15 patients (13%); among them, 10 disclosed exonic deletions. After a median follow up of 6 years, 21 patients (17%) experienced acute heart failure (AHF), 7 patients (6%) supraventricular arrhythmia, 3 patients (2.4%) ventricular tachycardia, 4 patients (3%) significant electrical disturbances. LBBB was significantly associated with cardiac events (OR = 12.7; 95%CI [3.78–42.7]; p <0.0001) and mortality (OR = 4.4; 95%CI [1.44–13.7]; p 0.009). Presence of residual dystrophin protein was not associated with significant less cardiac events. Age and LVEF were also predictive factors for cardiac events and mortality.

**Conclusion:**

LBBB is relatively frequent in DMD and is a major predictive factor for cardiac events and mortality. Presence of residual dystrophin protein was not associated with a lower incidence of cardiac events.

## Introduction

Duchenne muscular dystrophy (DMD) is an inherited myogenic disorder due to mutations in the dystrophin gene *DMD* on chromosome Xp21.1. Usually, DMD arises from out of frame DMD gene mutations which lead to the absence or the presence of very low amounts of dystrophin. It is the most common and one of the severe forms of muscular dystrophy and occurs in 1 / 5000 male births [1]. Cardiac and respiratory impairment are classical in this disease and strongly impact life expectancy [2, 3, 4]. Over the last few decades, the possibility to support pulmonary function with home mechanical ventilation (HMV) has radically improved the survival in DMD [5, 6, 7]. However, cardiac failure remains a classical complication that still affects survival in DMD. In the general population, left bundle branch block (LBBB) is classically associated with heart failure and with a poorer prognosis [8, 9]. Little is known about the impact of LBBB on prognosis in DMD patients. We designed this study to determine the prevalence of LBBB, the potential relationship between LBBB and genetic pattern and to assess predictive factors for acute cardiac events and mortality in adult DMD patients.

## Methods

### Study design

We retrospectively reviewed all the medical charts of DMD patients followed at the Home Mechanical Ventilation Unit of the Raymond Poincare University Hospital, a tertiary neuromuscular center (Garches, France), from 2006 to 2015. Neuromuscular patients are followed in the unit at least yearly to monitor their cardiac and respiratory functions. We included adult DMD patients (≥18 years). The first visit in the unit that included a cardio-respiratory assessment was considered as the baseline for the present study. For each patient, we collected clinical baseline, electrocardiogram (ECG), cardiac function and outcome data. Electrocardiograms have been interpreted blindly by two experienced cardiologists (AF/ON).

At each visit, details on intercurrent hospitalizations in other hospitals have been collected in the medical chart. The study was performed in compliance with the ethical principles formulated in the declaration of Helsinki and was approved by the internal review board (*comité de protection des personnes)* and the French regulatory board (*commission nationale de l'informatique et des libertés)*. The study was registered in ClinicalTrials.gov (identifier: NCT02501083)[10].

### Study endpoints

The aim of the study was to determine the prevalence of LBBB, the potential relationship between LBBB and genetic as well as dystrophin protein patterns, and the impact of LBBB and left ventricular ejection fraction (LVEF) on cardiac events and mortality in DMD. Cardiac events were defined as the onset of acute heart failure syndrome (AHF), cardiac arrhythmia (atrial fibrillation, atrial flutter, ventricular tachycardia, and ventricular fibrillation), significant conduction block (atrio-ventricular block type II or III, sino-atrial block type III) and vascular thromboembolic events. AHF was defined by the rapid onset or worsening of symptoms/signs of heart failure [11]. AHF diagnosis was made in our study for any patient that presented with symptoms of heart failure associated with congestion and left ventricular impairment [11].

### Genetic analysis

DMD diagnosis was based on *DMD* gene analysis that had been done prior to or was proposed during patient follow-up at the Home Mechanical Ventilation Unit of the Raymond Poincare University Hospital. Semi-quantitative fluorescent multiplex PCR using genomic DNA was used for detecting large exonic deletions and duplications in the *DMD* gene. Other types of mutations were detected by direct sequencing of the entire *DMD* gene exons or preceded by the analysis of muscle dystrophin mRNA by RT-PCR. For each patient, we recorded the mutation type and the involved gene exons; we also determined the most distal dystrophin domain theoretically involved by the DMD gene mutation and beyond that which the protein is truncated if any dystrophin is produced. Dystrophin protein is composed of several functional domains, i.e. the N-terminal domain, rod domain (composed of three subdomains separated by four hinges H1, H2, H3, and H4 which we respectively indicated as <H2, H2–H3, and >H3), cysteine-rich domain and C-terminal domain.

### Protein analysis

Muscle biopsy for dystrophin protein analysis was performed in 50 among the 121 patients prior or during their follow up in our center according to standard procedures [12]. Residual dystrophin amount was assessed using immunohistochemistry (IHC) and/or western blot (WB) from muscle sample. NCL-DYS1 antibody (recognizing dystrophin rod domain) and NCL-DYS2 antibody (recognizing dystrophin C-terminal) were used for this purpose [13].

### Cardiac function

ECG and echocardiographic data were collected from medical records. LBBB was defined by a feature of prolonged intra-ventricular conduction with QRS complex duration ≥120ms and QS or rS morphology in leads V1 and V2 [14]. Echocardiography was performed with an Acuson CV70 ultrasound Siemens device. Echocardiographic measurements were made and interpreted according to the guidelines published by the American Society of Echocardiography [15, 11]. Specifically, LVEF was obtained using M-mode methods. We also recorded quality of cardiac imaging and assessed intra-operator agreement for LVEF for the main study cardiologist (AF).

### Statistical analysis

Continuous variables were described by median ± interquartile range (IQR) and compared by Wilcoxon Rank Sum test; dichotomous or categorical variables were described by number of subjects and percentage and compared by Fisher’s exact test. Survival curves were estimated by the Kaplan-Meier method and then compared by the log-rank test; univariable logistic models allowed to estimate the strength of association based on the odds ratio (OR) with 95% confidence intervals (95%CI). Multivariable logistic regression model, including all variables selected as associated with outcome on the basis of a p-value of 0.05 or less was also performed. Bland-Altman plots were established providing the mean bias and 95% limits of agreement for the assessment of intra-operator variability regarding LVEF measurement. Statistical analysis was performed using R® software (http://www.R-project.org/).

## Results

### Patient characteristics and genetics finding at baseline

Baseline characteristics and genetics of the population are detailed in Table 1. A total of 121 patients have been included in our study. Age ranged from 18 years to 41 years [median 24]. All patients were wheelchair bound and lost ambulation before 13 years old, at an average age of 10 years old. Median pulmonary vital capacity (VC) was 12% [7; 19.5] of predicted values. Almost all patients were on HMV (95%). On genetic analysis, a definite *DMD* gene mutation was found in 119/121 patients (98.3%), among which 54% disclosed large exonic deletions and *1*2% large exonic duplications. Non-sense mutations were found in 12% of patients while other exonic point mutations and intronic mutations were found in 16% and 4% respectively. In 2 patients, no *DMD* mutation was found despite a total absence of dystrophin on IHC and WB. Residual dystrophin protein was found in 11 patients (9%) using the IHC and in 5 patients (4%) using WB.

**Table 1:** Clinical and genetic characteristics of the study population.

Parameter	N (%) or median [IQR]
N	121
Age (y)	24 [21; 28]
Age of ambulation loss (y)	10 [9; 11]
VC (% pred)	12 [7; 18]
ACE inhibitors	108 (90%)
Beta blockers	61 (51%)
Diuretics	12 (10%)
QRS duration (ms)	100 [87; 118]
LBBB	15 (13%)
RBBB	41 (34%)
LVEF (%)	47 [40; 55]
***Mutation type***	
Exonic deletion	66 (54%)
Exonic duplication	14 (12%)
Non-sense mutation	15 (12%)
Other exonic point mutation	19 (16%)
Intronic mutation	5 (4%)
No mutation	2 (2%)
***First disrupted dystrophin domain***	
N-Terminal	17 (15%)
Cystein-rich domain	4 (3%)
Rod domain	97 (82%)
<H2	11 (11%)
H2-H3	68 (70%)
>H3	18 (19%)
Cystein-rich domain	4 (3%)

IQR: Interquartile range. VC: pulmonary vital capacity. LVEF: left ventricular ejection fraction. LBBB: left bundle branch block. RBBB: right bundle branch block. Y: years. The “First disrupted dystrophin domain” indicates the first involved dystrophin protein domain that is disrupted by the *DMD* out of frame mutation, going from the N-Terminal, through the road domain (either before the Hing 2 or <H2, between the hinges 2 and 3 or H2-H3 or after the hinge 3 or >H3) to more distal mutations involving the cysteine rich domain.

### Cardiac function and LBBB

**Fig 1:**
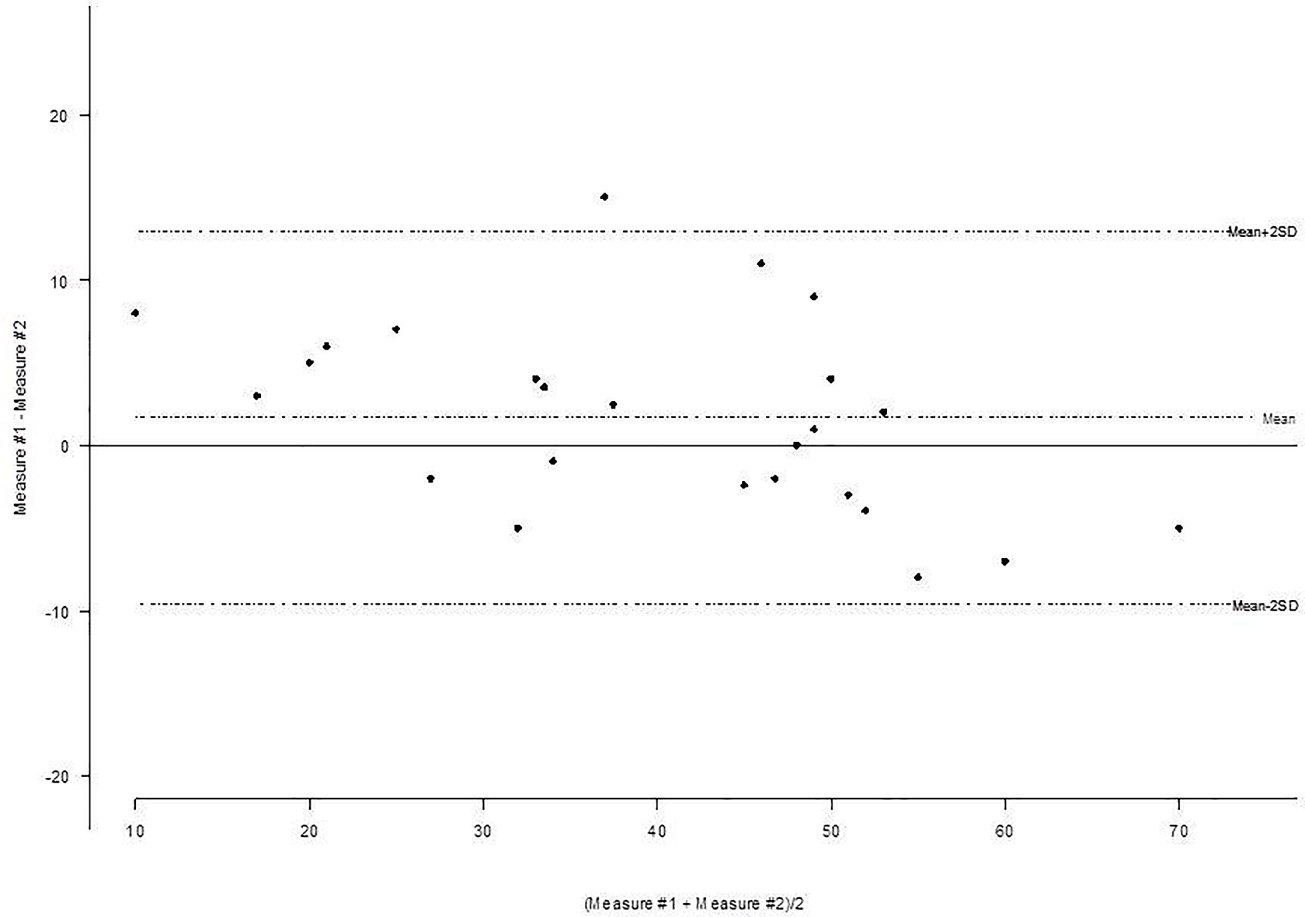
Bland-Altman plots for LVEF measurement (intra-observer variability analysis). LVEF: left ventricular ejection fraction.

All patients were in sinus rhythm and median QRS duration was 100 ms [87; 118]. 34% of patients exhibited a right bundle branch block (RBBB) whereas definite left bundle branch block (LBBB) was found in 13% (Table 1). The quality of echocardiographic scans was good in 91% of cases and focused on parasternal view axis for the evaluation of LVEF with a time movement (TM) mode. Median LVEF was 47% [40; 55]. Fig 1 reports the Bland Altman plot for intra-operator variability in the measurement of LVEF.

**Fig 2:**
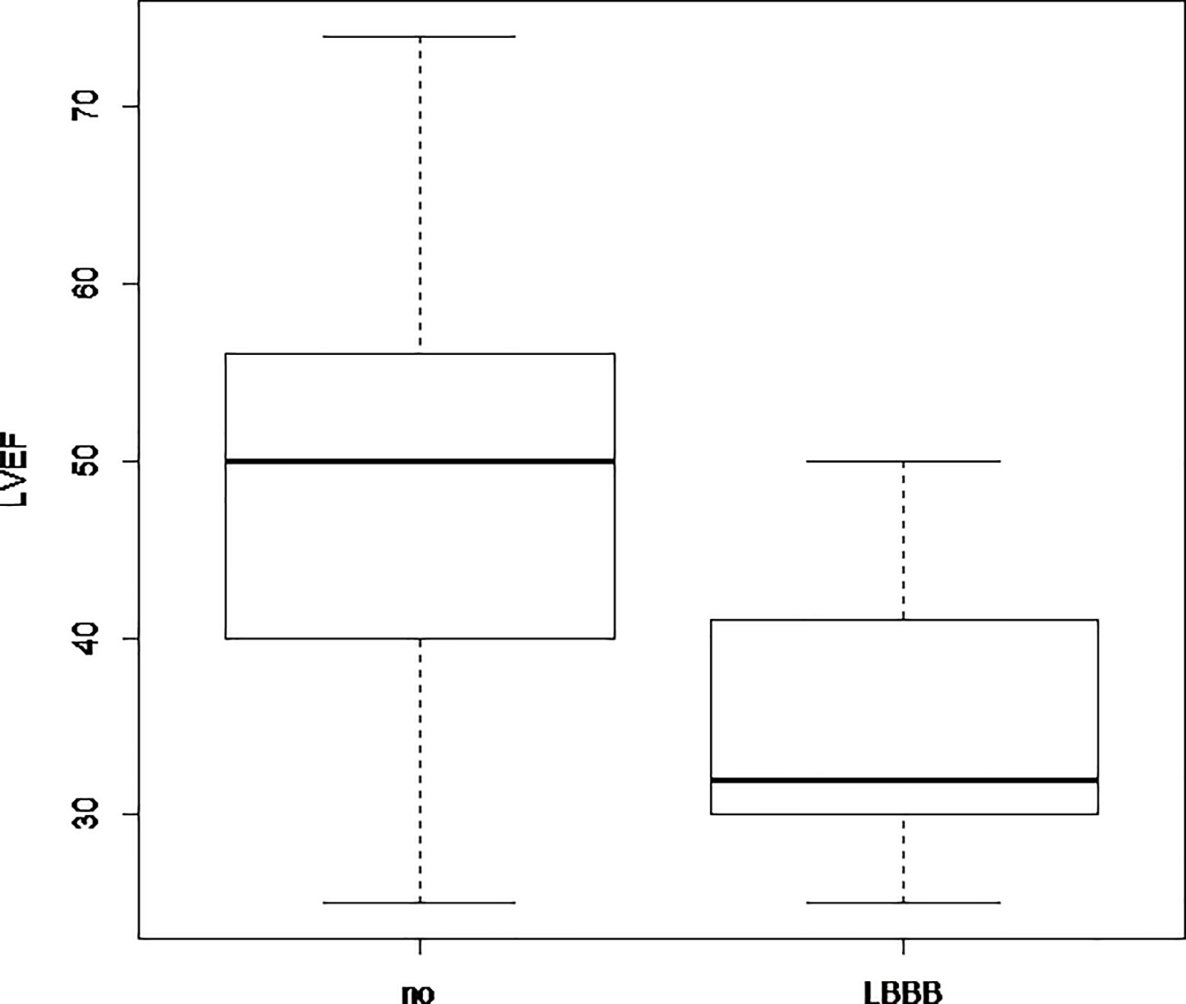
LVEF in DMD patients with LBBB vs patients without LBBB. LBBB: patients with left bundle branch block. No: patients without LBBB. LVEF (%): left ventricular ejection fraction.

We found an association between LVEF and LBBB, with significantly lower values of LVEF in patients with LBBB as compared to the others (Fig 2).

In patients with LBBB, median LVEF was 32 [IQR, 30–41] % *vs* 50 [IQR, 40–55.75] % in patients without LBBB (p< 0.001).

### Genetic-phenotype associations

Table 2 summarizes relationship between genetics and cardiac aspects while Table 3 indicates the associations between the presence of residual dystrophin protein using IHC and/or WB and cardiac events. Notably, 10 out of 15 patients with LBBB disclosed exonic deletion.

**Table 2:** Repartition of LBBB and LVEF regarding the type of mutation.

	Exonic deletions N = 66	Exonic duplications N = 14	Non-Sense mutations N = 15	Other exonic point mutations N = 19	Intronic mutations N = 5
***LBBB*** *(%)*	N = 10 (15%)	N = 2 (15%)	N = 1 (7%)	N = 1 (6%)	N = 1(20%)
***LVEF*** *(%)*	N = 65; 48 [40; 55]	N = 13; 45 [40; 50]	N = 15; 50 [45; 55]	N = 18; 47.5[35.5; 54.5]	N = 5; 36 [35; 54]

LBBB: left bundle branch block. LVEF: left ventricular ejection fraction. Data are expressed as median+-IQR or number (percentage).

**Table 3:** Residual dystrophin protein (IHC/WB) and cardiac events.

Presence of residual dystrophin protein (antibodies, IHC/WB)	No cardiac events (N = 96)	Cardiac events (N = 25)	*P value*	OR (95%CI)
DYS1 / IHC	10(30%)	1 (20%)	0.64	0.58 (0.06–5.8)
DYS2 / IHC	10(10%)	1 (4%)	0.34	0.36 (0.044–2.94)
DYS1 /WB	4(10%)	1 (17%)	0.61	1.85(0.17–20.0)
DYS2 / WB	4(4%)	1 (4%)	0.97	0.96(0.10–8.97)
*DYS1 and/or DYS2*, *IHC and/or WB*	12 (12%)	1 (4%)	0.30	0.29 (0.04–2.36)

IHC: immunofluorescence. WB: Western blot

We observed that among 13 patients who expressed residual amounts of dystrophin, cardiac events occurred only in one. However, no significant difference was found about cardiac events onset in the group with residual dystrophin protein (either by IF and/or WB) *vs* without residual dystrophin protein. Finally, we found no relationship between LBBB, cardiac events and the mutation location within the dystrophin protein (Tables 4 and 5).

**Table 4:** Mutation location and cardiac events.

First dystrophin disrupted domain	No Cardiac events (N = 96)	Cardiac events (N = 25)	P Value (Fisher)
**N-Terminal**	11 (13%)	6 (24%)	0.24
**<H2**	7 (8%)	4 (16%)	
**H2-H3**	57 (61%)	11 (44%)	
**>H3**	15 (16%)	3 (12%)	
**Cysteine rich domain**	3 (3%)	1 (4%)	

The “First disrupted dystrophin domain” indicate the first involved dystrophin protein domain that is disrupted by the *DMD* out of frame mutation, going from the N-Terminal, through the road domain (either before the Hing 2 or <H2, between the hinges 2 and 3 or H2-H3 or after the hinge 3 or >H3) to more distal mutations involving the cysteine rich domain.

**Table 5:** Mutation location and left bundle branch block.

First dystrophin disrupted domain	No (N = 103)	LBBB (N = 15)	P Value (Fisher)
**N-Terminal**	14 (14%)	2 (13%)	0.24
**<H2**	8 (8%)	3 (20%)	
**H2-H3**	61 (61%)	6 (40%)	
**>H3**	13 (13%)	4 (27%)	
**Cysteine rich domain**	4 (4%)	0	

The “First disrupted dystrophin domain” indicate the first involved dystrophin protein domain that is disrupted by the *DMD* out of frame mutation, going from the N-Terminal, through the road domain (either before the Hing 2 or <H2, between the hinges 2 and 3 or H2-H3 or after the hinge 3 or >H3) to more distal mutations involving the cysteine rich domain. LBBB: left bundle branch block.

### Cardiac events, LBBB and prognosis

The median follow-up lasted 6 years. The five-year cumulative incidence of cardiac events was 17.6% [95%CI: 10.3–25] and the five-year survival rate was 81.6% [95CI%: 74.4–89.5]. Among cardiac events, 21 patients (17%) experienced AHF, 7 patients (6%) supraventricular arrhythmia, 3 patients (2%) ventricular tachycardia and 4 patients (3%) significant electrical disturbances. Cardiac events among the 21 episodes of AHF were divided as follow: cardiogenic shock (6 events/21, 28%), left decompensated chronic heart failure (10 events/21, 47%), right and left decompensated chronic heart failure (5 events/21, 24%). One patient experienced an episode of acute ischemic stroke and an asymptomatic left intraventricular thrombus was found in one patient.

**Fig 3:**
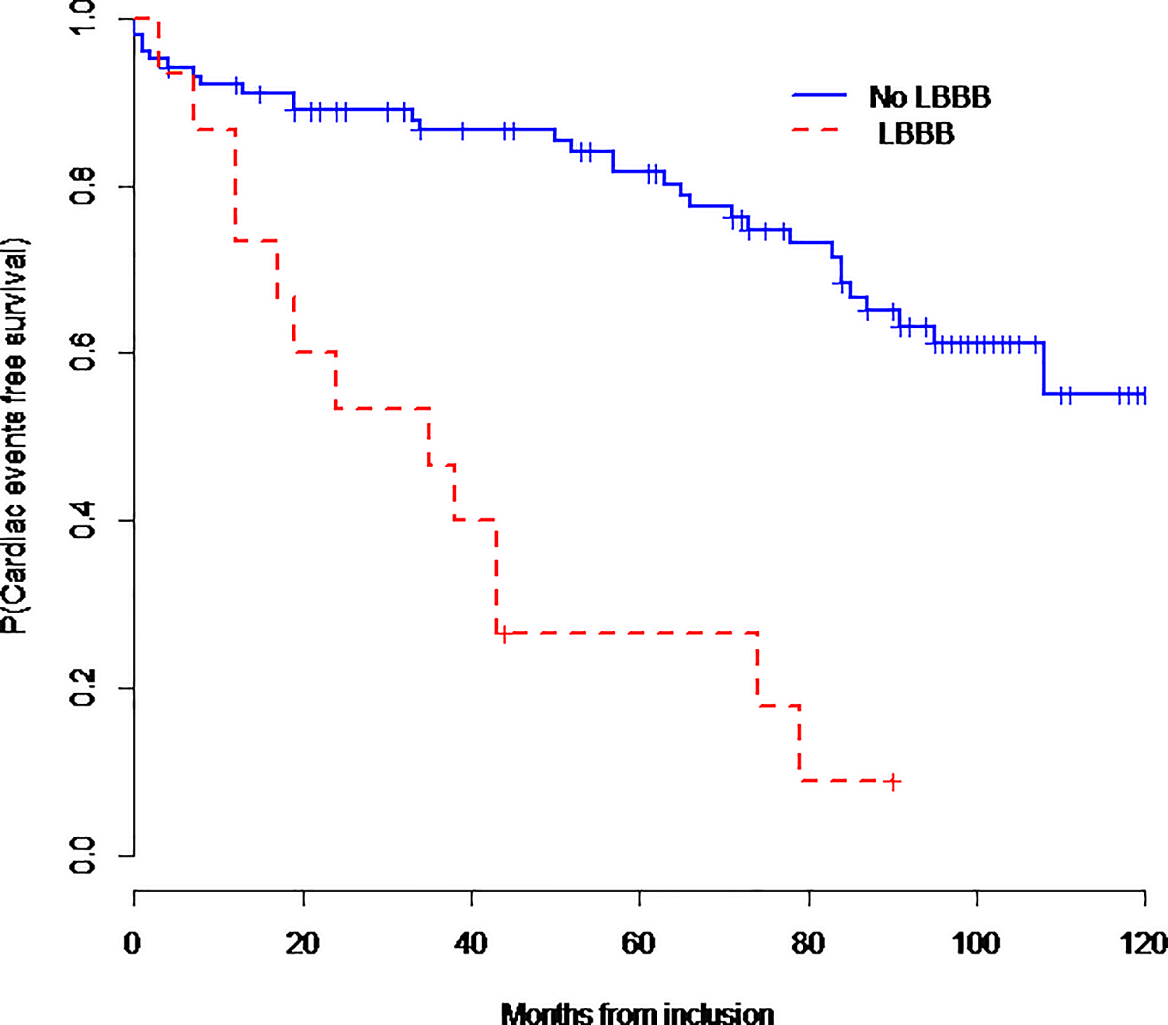
Cardiac events free survival (EFS) according to the presence or the absence of LBBB.

**Fig 4:**
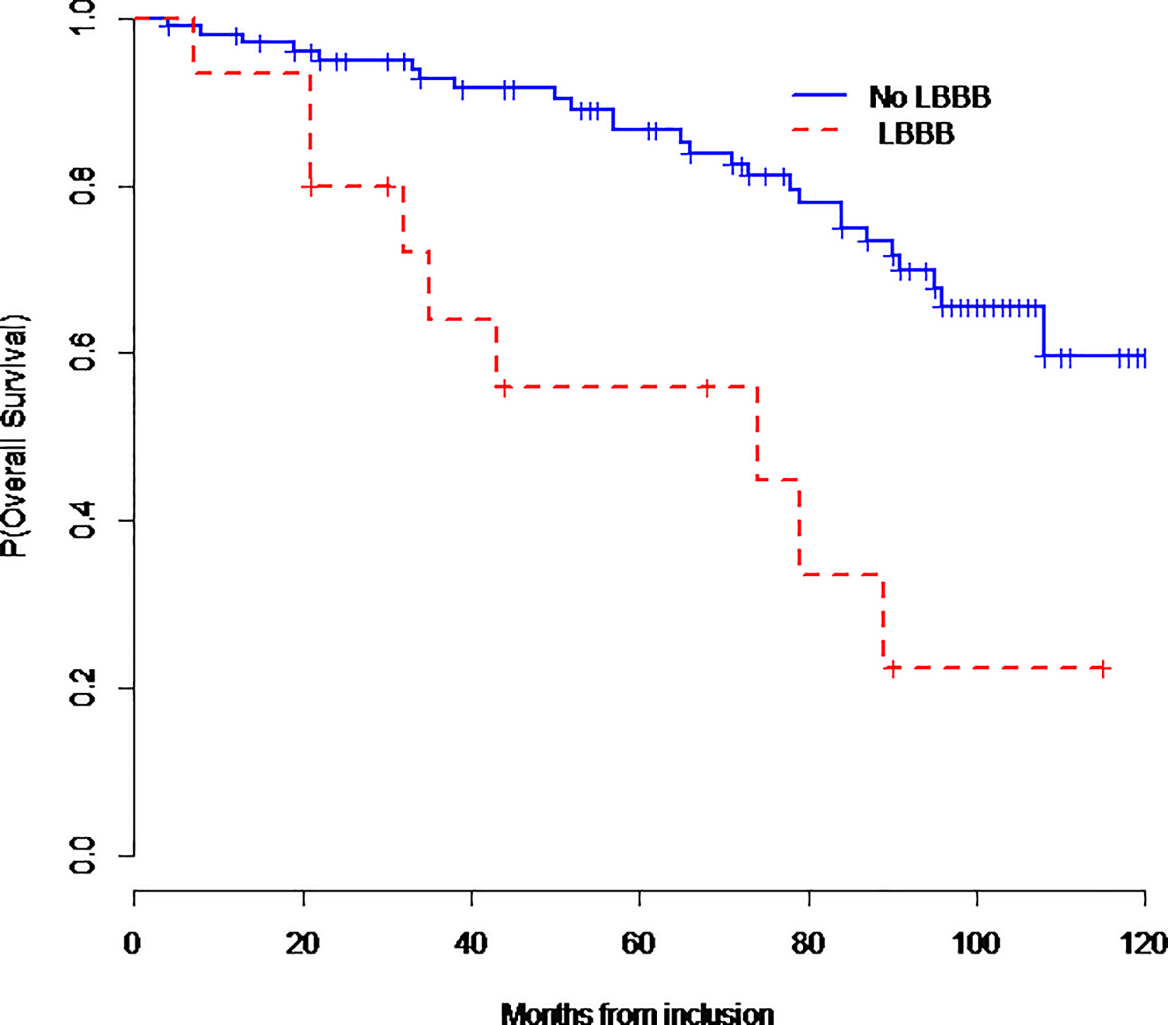
Global survival curve according the presence or the absence of LBBB.

Table 6 summarizes cardiac predictive factors including LBBB. Fig 3 and Fig 4 show respectively cardiac events-free survival and overall survival curves according to the presence or absence of LBBB. Four patients with significant electrical disturbances benefited from pacemaker implantation device and two patients with ventricular tachycardia benefited from implantable cardioverter defibrillator (ICD) device implantation.

**Table 6:** Predictive factors associated with cardiac events using univariate analysis.

Factors	No cardiac events (N = 96)	Cardiac events (N = 25)	p value	OR (95%CI)
Age (y)	23 [21; 27]	26 [22; 32]	0.045	1.08(1.00–1.17)
Age of ambulation loss (y)	10 [8.5; 11]	11 [9.5; 11.75]	0.25	1.22(0.87–1.70)
diuretic	3 (3%)	9 (36%)	<0.0001	16.9(4.12–69.2)
QRS duration (ms)	96 [86; 106]	121 [88.5; 134]	0.007	1.04(1.01–1.07)
LVEF (%)	50 [40.5; 56.75]	35.5 [30; 46.25]	0.0001	0.90(0.86–0.95)
LBBB (%)	5 (5%)	10 (42%)	<0.0001	12.7(3.78–42.7)
RBBB (%)	35 (37%)	6 (25%)	0.28	0.57(0.21–1.57)

*Data are expressed as median+-IQR or number (percentage)*. LVEF: left ventricular ejection fraction. LBBB: left bundle branch block. RBBB: right bundle branch block.

LBBB was significantly associated with cardiac events (OR = 12.7; 95%CI [3.78; 42.7]; p<0.0001) and mortality (OR = 4.44; 95%CI [1.44–13.7]; p = 0.009). Lower LVEF was also associated with cardiac events (OR = 0.90; 95%CI [0.86; 0.95], p = 0.0001) and mortality (OR = 0.96; 95%CI [0.92; 0.99], p = 0.023). However, once LBBB is used to predict cardiac events, the additional predictive value of LVEF was no longer statistically significant.

With multivariable logistic regression model, three variables were selected as adding to each other predictive information, namely age, use of diuretics, and LBBB (Table 7).

**Table 7:** Predictive factors for cardiac events using multivariable logistic regression.

	*OR (95%CI)*	*p-value*
Age, years	1.12 (1.015–1.24)	0.024
Diuretic	14.5 (3.0–70.2)	0.0009
LBBB	7.10 (1.84–27.5)	0.0047

LBBB: left bundle branch block

20 deaths have been documented among the 35 events. 60% were related to cardiac disease where as 40% were related to respiratory causes. The main causes of cardiac deaths were end stage heart failure (10 /12, 84%) and rarely cardiac arrest (2 /12, 16%) without arrhythmia documentation. The two cardiac arrests involved 2 patients with chronic heart failure and severe respiratory impairment requiring permanent invasive ventilation (24h/24h).

In this context of end stage heart failure, a cardiac resynchronization therapy (CRT) device was offered to 2 patients (1 with CRT-Pacemaker and 1 with CRT-Defibrillator). We noticed that among the 14 patients with LBBB and heart failure, cardiac symptoms, cardiac function and survival rate improved only in the 2 patients with implanted cardiac device. Conversely, in the 12 other patients with LBBB and without CRT device implantation, mortality rate was higher (6 patients /12), despite medical therapy.

## Discussion

Our results allowed us to define the accurate prevalence of LBBB, i.e. 13% in this large series of 121 adult DMD patients on HMV, 98% of them carrying a definite *DMD* gene mutation. We also observed that LBBB is a significant predictive factor for cardiac events, whilst patients with residual dystrophin protein did not show a decreased incidence of cardiac events.

Cardiomyopathy has a high prevalence among DMD patients, affecting the left ventricle and leading to chronic congestive heart failure and heart rhythm disturbances [16, 17]. Even if angiotensin-converting-enzyme (ACE) inhibitors and beta-blockers have positive impact on heart function in DMD [18, 19], cardiomyopathy remains a challenging problem and a source of mortality in this population. Indeed, DMD is due to mutations in *DMD* gene encoding a protein located on the inner side of the skeletal and cardiac muscle cells. The *DMD* gene is one of the largest genes known in humans (2.3Mb of genomic DNA). Depending on the conservation of the translational reading frame, these mutations usually lead to DMD in case of out of frame mutations and absence of dystrophin. Dystrophin protein has a major structural role in muscle, as it links the internal cytoskeleton to the extracellular matrix. The lack of dystrophin leads to gradual fiber damage and membrane leakage, resulting in a progressive muscle wasting and weakness of variable distribution and severity [20].

In dilated cardiomyopathy, progression of heart failure is often associated with LBBB [21]. In a recent French study that included patients with acute heart failure syndrome, the authors reported a prevalence of LBBB at 16% and the presence of LBBB was independently associated with higher 1-year mortality [22]. In our study, LBBB was present in 13% of patients. We found that the presence of LBBB was significantly associated with higher incidence of cardiac events (OR = 12.7, p<0.0001) and higher long-term mortality. Likewise, in the general population, the presence of a LBBB is associated with an increase of cardiac morbidity and mortality and seems to reflect progressive degenerative process that affects heart. Our study found an association between LVEF and LBBB, with significantly lower values of LVEF in patients with LBBB. However, once LBBB is used to predict cardiac events, the additional predictive value of LVEF was no longer statistically significant.

Regarding electrical cardiac events, the incidence of supraventricular and ventricular arrhythmia as well as conduction disturbance was relatively low in our study, in line with data reported by others [23, 24]. In our study, AHF was present in 17% of patients, supraventricular arrhythmia in 6%, ventricular tachycardia in 2% and significant electrical disturbances in 3%. Perloff *et al* [23] reported atrial flutter in 5% of patients and sinus pause in 5% of patients in a study that included 20 patients. Seven percent of patients disclosed ventricular tachycardia in the study by Corrado *et al* [25], reaching 16% in a study including DMD and Becker muscular dystrophies [24]. Moreover and in accordance with our findings, LVEF has been reported to be a predictive factor for mortality in DMD [24, 25].

In DMD, studies about genotype-phenotype correlations are challenging since dystrophin is usually absent and proteins are truncated. Heart impairment has been reported to be present mainly in patients with mutation involving exons 12 to 17 in DMD patients [26]. Also, deletions in the hotspot region (exons 45–55) tend to be associated with a milder phenotype [26]. Residual dystrophin level was not previously investigated. In our study, we investigated the potential effect of any residual dystrophin expression as well as the mutation position within the dystrophin subdomains. Even if patients with residual dystrophin protein are more likely to disclose less cardiac events, no significant difference with those patients without residual dystrophin was observed. A residual dystrophin protein was not associated with lower incidence of cardiac events. Moreover, there is no association between mutation location and the occurrence of cardiac disease and cardiac events.

Finally, in the last two decades, HMV has improved outcomes and survival rate in neuromuscular disorders [5, 6, 7, 27, 28, 29]. In our study, the five-year survival rate was 81.6%, which is similar to other studies (73% in the study by Kohler) [30]. However, the presence of a complete LBBB is associated with a significantly increased mortality (OR 4.44, p = 0.009). In our study, cardiac deaths are mainly due to end stage heart failure rather than rhythmic events. In the 2 patients with heart failure and LBBB, the implantation of CRT device has improved symptoms and survival rate. Even if, this technique has been performed in a small number of patients as a rescue therapy for end-stage disease, these findings suggest a potential interest of performing CRT in DMD patients with LBBB and LV dysfunction. Indeed, in the general population, an increase of QRS duration is associated with worse prognosis in patients with acute heart failure [31]. In fact, in the general population, the ESC 2016 guidelines [11] recommend a CRT for symptomatic patients with heart failure in sinus rhythm with a QRS duration ≥150ms and LBBB and with a LVEF≤35% despite optimal medical therapy in order to improve symptoms and to reduce morbidity and mortality (class I, level A). Also, a CRT is recommended for symptomatic patients with heart failure in sinus rhythm with a QRS duration of 130-149ms and LBBB and LVEF ≤35% despite optimal medical therapy in order to improve symptoms and to reduce morbidity and mortality (class I, level B) [11]. We have previously demonstrated that this procedure is safe and may improve LV function in DMD patients [32] but needs a careful selection of the patients because of technical difficulties [10]. In our study, two DMD patients with heart failure and LBBB improved their symptoms and heart function after a CRT device implantation, whereas mortality remains higher in DMD patients with heart and LBBB without CRT device implantation.

CRT should be considered in DMD patients with heart failure based on the prevalence of LBBB reported in our study and the previous work suggesting the usefulness of CRT.

### Limitations of the Study

The first limit is about the echocardiographic analysis in non-ambulant and ventilated DMD patients. Since chest deformities, lung inflation with ventilation, limited mobility and scoliosis are causes of technical difficulties impairing echocardiographic exploration [12], it is not easy to keep DMD tracheotomised patients in the left decubitus position, rendering it difficult to obtain a two–dimensional echocardiography recording from an apical view. A parasternal M mode echocardiography is easy to perform and it has been reported a good reproducibility using this technique in patients with muscular dystrophies [33].Because of its retrospective nature, we have not been able to perform inter-observer variability study in the patients involved in our study, however intra-observer variability was low and the quality of imaging proved to be good. Our study was mono-centric and retrospective and the results may be hampered by the limited sample size and the recruitment specificities of our unit. Finally, this study was not designed to assess the efficacy of CRT in DMD patients with LBBB.

### Future perspective

Heart failure remains a significant cause of morbidity and mortality in patients with DMD. As reported in our study, prognosis depends on LVEF and LBBB onset. Cardioprotective drugs (ACE inhibitors) can be initiated in young DMD patients to delay the onset of cardiac deterioration [18]. In patients with cardiomyopathy, ACE inhibitors and beta-blockers remain the cornerstone drugs used in practice, sometimes associated with mineralocorticoid receptor antagonists. The onset of LBBB worsens prognosis and probably needs a more aggressive approach that may include CRT. Indeed, this instrumental therapy should be discussed in DMD patients with heart failure based on the prevalence of LBBB reported in our study and the previous work indicating the usefulness of CRT. Future multi-centric prospective studies may be helpful to better characterize prognostic factors in DMD and to assess potential indications for CRT device in DMD.

In parallel to this potential invasive approach, research focuses currently on non-invasive innovative approaches including exon skipping, CRISPR/Cas [34]and other gene therapy techniques [35].

## Conclusion

LBBB is frequent in adult DMD and is associated with poor prognosis. LBBB adds to impaired LVEF for cardiac prognosis evaluation. Presence of residual dystrophin protein seems not to be associated with a lower risk of heart events.

We suggest LBBB and rhythmic events to be screened by annual ECG and Holter ECG in non-ambulant DMD patients with heart dysfunction.

CRT has <<potential>> efficacy for patients with DMD, LBBB and associated with heart failure.

## Supporting information

S1 File DMD PLOS ONE supplemental data. (XLSX)
